# Pre-donation deferral patterns in blood donor recruitment in a tertiary care hospital

**DOI:** 10.12669/pjms.42.(ICON26).15700

**Published:** 2026-04

**Authors:** Poonam Devi, Neelum Mansoor, Saba Jamal

**Affiliations:** 1 Dr. Poonam Devi, MBBS., Clinical Laboratories and Blood Transfusion Services, Indus Hospital and Health Network, Karachi, Pakistan; 2 Dr. Neelum Mansoor, FCPS., Clinical Laboratories and Blood Transfusion Services, Indus Hospital and Health Network, Karachi, Pakistan; 3 Dr. Saba Jamal, Diplomate American Board of Hematology, Clinical Laboratories and Blood Transfusion Services, Indus Hospital and Health Network, Karachi, Pakistan

**Keywords:** Blood donation, LMIC, Permanent deferral, Temporary deferral, Voluntary donors

## Abstract

**Objectives:**

This study analyzes pre-donation deferral patterns in blood donor recruitment at a tertiary care hospital in Pakistan to improve blood supply management.

**Methodology:**

A cross-sectional analysis was conducted on 6,039 potential blood donors aged 18–60 years at the Indus Hospital Blood Center, Karachi, from April-June 2023. Physical examinations and clinical evaluations determined the donor’s eligibility. Deferral rates and reasons were analyzed using descriptive statistics.

**Results:**

The average deferral rate was 27.4%, with 97.7% temporary and 2.3% permanent deferral. Majority donors were first-time (92.3%) and male (93.3%). Common temporary deferral reasons were anemia (29.0%), ill-feeling (21.9%), and antibiotic intake (9.8%), while Hepatitis C (23.7%) and Hepatitis B (21.1%), and chronic respiratory disease (18.4%) were permanent.

**Conclusion:**

The study reveals challenges in blood donor recruitment, including high first-time donors and gender disparity. Targeted measures may help address risk factors and improve donor retention, strengthening blood transfusion services in resource-poor settings, impacting public health in Pakistan and other low- and middle-income countries (LMICs).

## INTRODUCTION

Blood transfusion is an integral part of modern healthcare systems and has a vital role in medical and surgical interventions. A safe and adequate blood supply is necessary for the management of emergencies, chronic diseases, and complex medical procedures.^1^ This is challenging in developing countries like Pakistan, where blood donation rates are lower than in developed nations.^2^

Blood donor recruitment involves several factors and strict screening to prevent risks for both donors and recipients. Pre-donation screening excludes people with increased risk of adverse effects from donation or whose blood may present a risk to potential recipients.^3^ This screening includes questionnaires, physical examination, and laboratory tests, based on local regulations and available resources. Blood donor recruitment and pre-donation screening are complex in LMICs like Pakistan due to limited resources, inadequate infrastructure, and sociocultural factors. Pakistan’s blood transfusion system faces issues such as a fragmented blood banking structure, dependence on family replacement donors, and a low voluntary non-remunerated blood donation (VNRBD).^4^

Risk factors can either be temporary or permanent deferrals based on the nature and duration. Temporary deferrals occur for specific periods due to low hemoglobin levels, recent infections, or some specific medications, while permanent deferrals arise due to chronic health conditions or high-risk behavior.^5^ Both types of deferrals affect blood supply management and donor retention strategies.^6^

Research from developed countries on blood donor deferral trends provides benchmarks for comparison and potential areas of intervention for possible improvement. A large US study showed 13.4% deferral rate primarily due to low hemoglobin.^7^ Deferral rates and causes vary between developed countries and LMICs due to differences in population health, screening practices and resources.^8^

Understanding pre-donation deferral trends in a tertiary care hospital in Pakistan carries several implications. Although Pakistani literature has reported rates of donor delays and medical causes such as anaemia or infection, important gaps remain. 9 Our study addresses those gaps by using a detailed locale-tailored donor history questionnaire that captures context-specific factors such as medication use, dental treatment, and needle exposure, often overlooked in earlier research. This approach provides a more accurate and locally relevant assessment of deferral reasons. The deferral rates may be lowered via the identification of common reasons and directing interventions to reduce modifiable risk factors. It can provide a base for stronger donor education and recruitment strategies targeting the local populations’ unique needs. It may also optimize screening protocols to maximize the donor pool while ensuring blood safety. Deferral trends may also be useful in assessing the health status of the donor population as proxy public health indicators. This study examines the pre-donation deferral trends in blood donor recruitment in a tertiary care hospital in Pakistan. Although patterns of donor deferrals are well researched in other LMICs,^10^ there is limited data on Pakistan. Local research has focused mainly on reasons for deferral in the laboratory, whereas little attention has been paid to behavioral and socio-cultural aspects affecting donors’ eligibility.^11^ The blood-donation system in Pakistan is experiencing distinct challenges that encompass reliance on replacement donors, low female participation, prevalence of injectable medications, and inconsistent screening; these gaps persist.^12^ This study addresses them through a comprehensive donor history questionnaire designed to capture population-specific risk factors, providing a deeper understanding of deferral patterns in Pakistan. Our results will contribute to the existing knowledge of blood donor management in resource-limited settings through analysis of these trends and comparison with other LMIC and developed countries.

## METHODOLOGY

The study population comprised of potential blood donors. This cross-sectional study was carried out from 1^st^ April, 2023, to 30^th^ June, 2023, and included the donors aged 18-60 years, excluding children, pregnant females, and nursing mothers, to assess donor deferral rates and causes at TIHBC. Based on the prevalence of donor deferral of 12.2%^13^ (margin of error-5%; CI-95%), the sample size was calculated to be 6039. The non-probability consecutive sampling technique was used. Data collection involved physical examination (presence of jaundice, blood pressure, pulse, temperature, and weight) and clinical evaluation (hemoglobin). A predesigned data sheet categorized donor deferral as temporary (unable to donate for a limited time period) or permanent (ineligible to donate blood in their lifetime).

### Ethical Approval:

It was obtained from the Institutional Review Board approval (IHHN_IRB_2025_04_008; dated May 12, 2025),

### Statistical analysis:

Data was analyzed by using SPSS v26.0. Median (IQR) was computed for quantitative variables based on their normality, while frequencies and percentages were reported for categorical variables. Association between independent variables and deferral type was assessed using the chi-square/Fisher-Exact test, with a p-value of ≤ 0.05 considered significant.

## RESULTS

Based on the inclusion criteria, a total of 6039 donors were enrolled in the study. The study participants were predominantly male, i.e., 93.3% (n=5638). The demographic, physical, and clinical information is shown in Table-I. In the present study, first-time donors were the most frequent, i.e., 92.3% (n=5573). In terms of the type of donors, voluntary donors were observed to be the majority, i.e., 71.5% (n=4319) (Table-I).

**Table-I:** Demographic, physical, laboratory, and donor information of study participants (N=6039).

Parameter	n (%)
***Demographics***	
***Gender ^Φ^***	
M/F	5638 (93.3)/403 (6.7)
Age, years^Δ^	35.0 (28.0-42.0)
***Physical findings***	
Weight, kg^Δ^	68.0 (63.0-74.0)
Pulse, bpm^Δ^	83.0 (76.0-88.0)
Temperature, F^Δ^	98.6 (98.6-98.6)
Systolic blood pressure, mmHg^Δ^	120.0 (110.0-130.0)
Diastolic blood pressure, mmHg^Δ^	80.0 (70.0-85.0)
***Presence of jaundice^Φ^***	
Yes	4 (0.1)
No	6037 (99.9)
***Laboratory findings***	
Haemoglobin, g/dl^Δ^	15.0 (13.0-16.0)
***Donor information ^Φ^***	
First time Repeat	5573 (92.3) 468 (7.7)
Voluntary donors Exchange donors	4319 (71.5) 1722 (28.5)

Φ-n (%); Δ-Median (IQR).

**Fig.1:**
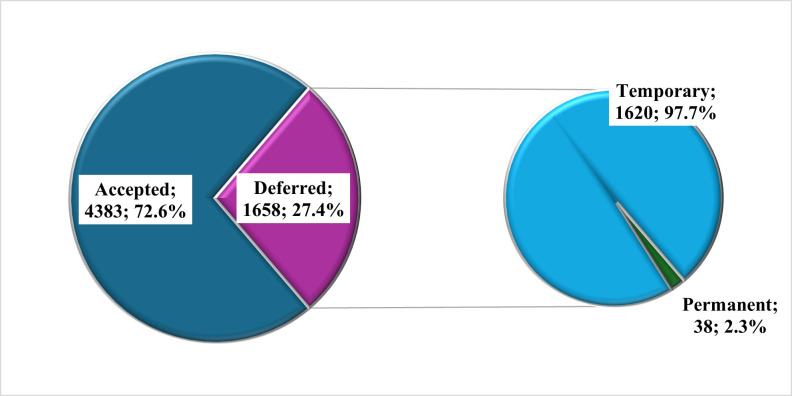
Frequency and type of deferral.

**Fig.2:**
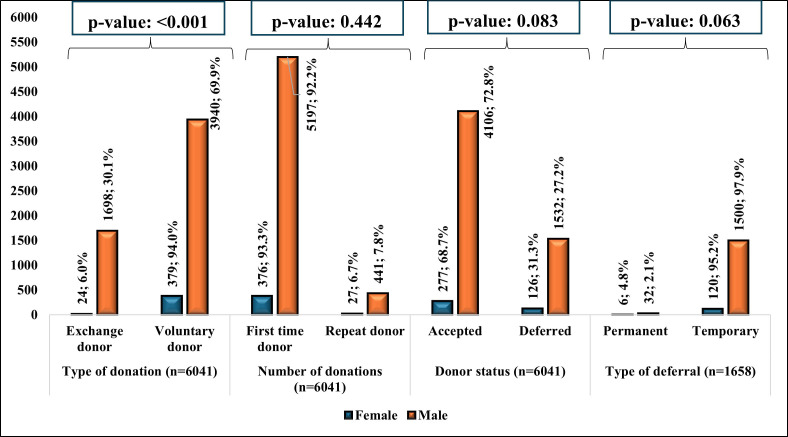
Frequency of temporary and permanent deferrals among male and female donors.

Of the total 6039 people who came for donation, 27.4% (n=1658) were deferred. Of the deferred patients, the majority were temporarily deferred (n=1620; 97.7%) while the remaining were permanently deferred (n=38; 2.3%) (Fig.1). The distribution of the male and female donors based on the type of deferral demonstrated an insignificant association (p-value: 0.063), as shown in Figure 2. The most frequent reasons for the temporary deferral of the donors included low hemoglobin (<13 g/dl) (n=470; 29.0%), sense of ill-feeling (n=354; 31.4%), and antibiotic intake (n=156; 9.6%). For permanent deferral, the most common reasons included being positive for Hepatitis C (n=9; 23.7%), Hepatitis B (n=8; 21.1%), and chronic respiratory disease (n=7; 18.4%).

The low level of hemoglobin, sense of ill-feeling, antibiotic intake, positive for Hepatitis B, positive for Hepatitis C, positive for HIV, presence of chronic respiratory disease, cardiac issues, epilepsy, diabetes mellitus, and hypertension were found to have a significant association with the type of deferral (p-value <0.05) (Table-II).

**Table-II:** Association of various reasons of deferral with the type of deferral

Reason	Type of deferral		p-value
	Permanent (n=38)	Temporary (n=1620)	
Haemoglobin (<13 g/dl)	0	470 (29.0)	<0.001^¥^*
Not feeling well	0	354 (21.9)	0.002^β^*
Intake of antibiotic	0	158 (9.8)	0.044^¥^*
Dental work (within the last three months)	0	85 (5.2)	0.257^¥^
Have cupping (hijama) performed on oneself	0	45 (2.8)	0.623^¥^
Sexual relations with an HIV, Hep B, or Hep C positive person	0	21 (1.3)	1.000^¥^
Malaria	0	17 (1.0)	1.000^¥^
Intake of medications	0	15 (0.9)	1.000^¥^
Exposure to viral hepatitis	0	11 (0.7)	1.000^¥^
Donation (within the last two months)	0	9 (0.6)	1.000^¥^
Hepatitis C virus	9 (23.7)	0	<0.001^¥^*
Hepatitis B virus	8 (21.1)	0	<0.001^¥^*
Chronic respiratory disease	7 (18.4)	0	<0.001^¥^*
Surgery (Past six months)	0	7 (0)	1.000^¥^
Transfusion-transmitted infections	0	6 (0.4)	1.000^¥^
Cardiac issues	4 (10.5)	0	<0.001^¥^*
Jaundice	0	4 (0.2)	1.000^¥^
Body piercing (past three months)	0	3 (0.2)	1.000^¥^
HIV	3 (7.9)	0	<0.001^¥^*
Vaccination (past three months)	0	3 (0.2)	1.000^¥^
Epilepsy	3 (7.9)	0	<0.001^¥^*
Diabetes Mellitus	3 (7.9)	0	<0.001^¥^*
Tuberculosis	0	3 (0.2)	1.000^¥^
Transfusion	0	2 (0.1)	1.000^¥^
Tattoo (past three months)	0	2 (0.1)	1.000^¥^
Chikungunya virus	0	2 (0.1)	1.000^¥^
Hypertension	1 (2.6)	0	0.023^¥^*
Dengue fever	0	1 (0.1)	1.000^¥^
Measles/Rubella	0	1 (0.1)	1.000^¥^
Typhoid	0	1 (0.1)	1.000^¥^
Hepatitis A virus	0	1 (0.1)	1.000^¥^
Pregnancy	0	1 (0.1)	1.000^¥^

¥ - Fisher-Exact test; β - Pearson’s Chi-Square test;

* - p-value statistically significant at < 0.05.

## DISCUSSION

The study found a predominance of male donors (93.2%) consistent with local studies.^9^,^12^ A systematic review of thirty-three articles by Ehsan et al.^11^ spanning from 2010–2020 reported male representation ranging from 95%–100% in Pakistan. This disparity exists in other LMICs, like India, with 95.6% male donors.^14^ Female blood donors are under-represented in Pakistan because of iron deficiency and health reasons. Research indicates a high prevalence of iron deficiency among female donors, necessitating screening and supplementation.^15^ Women are barred from recruitment, and socio-cultural factors. Even with increased blood drives in urban regions, the rate of female donors is still low, with males controlling the collections.^16^

The study observed 92.3% first-time donors, comparably higher than other local study, where Nadeem et al. observed 60.0%.^17^ The high percentage of first-time donors is a common occurrence in LMICs, which could be attributed to various reasons. The recruitment activities of the donor organizations are usually tailored to attract new donors rather than retaining repeat donors. The primary hindrances to retention are fear and anxiety about donation because a large proportion of the donors fail to donate again after having adverse reactions. Lack of knowledge of the donation opportunity does not allow for frequent donation. Low rates of repeat donations are caused by time and inconvenient location. Donor deferral is caused by medical disqualification. Absence of peer support lessens the desire to make repeat donations. Lack of proper donor tracking systems artificially increases the number of first-time donors in various sites.^11^,^16^ In contrast, in developed countries, Shaz et al. reported only 19% of first-time donors in the United States. 18

The study found 71.5% voluntary donors, far exceeding other Pakistani reports of 15% to 53%.^9^ The increase approaches the WHO recommended practices, though still behind the high-income countries, where the WHO reports 79 countries with >99% voluntary donation, with 38 being high-income.^19^

The current study reported a 27.4% deferral rate with 97.7% temporary and 2.3% permanent deferrals. For temporary deferral, anemia (29%; 470/1620) was the most common cause. A local study found a 12.2% overall deferral rate, with 91.1% being temporary, primarily due to anemia (30.8%).^13^ Studies from other LMICs showed similar trends, with India reporting 16.0% deferral rate and anemia as a significant cause (28.3%).^20^ Common patterns across LMICs can help plan targeted interventions for reducing deferral rates and improving donor management. Developed countries show different deferral reasons, with Custer et al.^21^ reporting travel to malaria-endemic areas as the main cause in the U.S. In this study, the high rate of deferral owing to “sense of ill feeling” (31.4%) is significant and requires investigation. Hepatitis C (23.7%) and Hepatitis B (21.1%) were the commonest reasons for permanent deferral in the present study, aligned with the findings of Saba et al.^9^ However, a local study by Taj et al.^22^ reported Hepatitis C (42%) and syphilis (35%) as the main causes. On the contrary, in developed countries like the U.S., hypertension and diabetes were the common reasons.^21^ The study found a higher deferral rate in females (31.3%) than males (27.2%), though not statistically significant. This significant gender disparity was also reported by local (Jamal et al.^23^ (54% vs. 21%)), regional (Narayanan et al.^24^ (47.9% vs. 16.0%)) in India, and in developed countries (Zou et al.^25^).

Over 92% were first-time donors, indicating a need for strategies to encourage repeat donations. Donor retention programs successful in developed countries can be adapted for the Pakistani context. While 71.5% of participants were voluntary donors, this could improve blood donation rates through public awareness campaigns of voluntary donation. The high deferral rate due to low hemoglobin suggests the need for nutritional interventions and pre-donation hemoglobin screening. Hepatitis B and C remain significant reasons for permanent deferral, highlighting the importance of robust screening and public health measures. Gender disparity requires targeted interventions to promote female blood donation.

### Limitations:

The data retrieval from a single blood center, limited the generalizability. The high male donor proportion may under-represent female donor characteristics. The cross-sectional design prevents establishing causal relationships. Some deferral reasons may be biased by self-reported health information. The study did not investigate the reasons for high first-time donor rates or explore donor retention strategies in Pakistan.

## CONCLUSION

The study shows positive trends in Pakistani blood donation practices, particularly an increase in voluntary donors, though improvements are needed. Donor demographics and deferral causes align with other LMICs but differ from developed countries. Further research is needed to understand the high first-time donor rate and ’sense of ill-feeling’ deferrals unique to the Pakistani context. These findings can improve blood donation practices in Pakistan and other LMICs with comparable challenges.

### Authors Contribution:

**PD:** Concept, analyzed data, and prepared the draft.

**NM SJ:** Study design, reviewed content, and approved final version

All authors have read the final version and are responsible and accountable for the accuracy and integrity of the work.
